# The Accessibility of YouTube Fitness Videos for Individuals Who Are Disabled Before and During the COVID-19 Pandemic: Preliminary Application of a Text Analytics Approach

**DOI:** 10.2196/34176

**Published:** 2022-02-15

**Authors:** Shevali Kadakia, Catherine Stratton, Yinfei Wu, Josemari Feliciano, Yetsa A Tuakli-Wosornu

**Affiliations:** 1 Department of Computing and Mathematical Sciences California Institute of Technology Pasadena, CA United States; 2 Department of Chronic Disease Epidemiology Yale School of Public Health New Haven, CT United States; 3 Department of Biostatistics Yale School of Public Health New Haven, CT United States; 4 Department of Physical Medicine and Rehabilitation University of Pittsburgh Medical Center Pittsburgh, PA United States

**Keywords:** persons with disabilities, disability, exercise, physical activity, digital health, YouTube, accessibility, fitness, COVID-19, text analysis, social media, video

## Abstract

**Background:**

People with disabilities face barriers to in-person physical activity (PA), including a lack of adaptive equipment and knowledgeable instructors. Given this and the increased need for digital resources due to widespread COVID-19 lockdowns, it is necessary to assess the accessibility of digital fitness resources for people with disabilities. To investigate whether YouTube fitness content creators have made videos accessible to people with disabilities would be informative about access to PA during COVID-19 and could also provide insight into the feasibility of individuals who are disabled relying on YouTube for PA in a post–COVID-19 world.

**Objective:**

This study aims to ascertain if disability-friendly PA videos on YouTube are accessible through searching general fitness terms and whether a change in the availability of accessible fitness resources for people with disabilities occurred on YouTube between before and during the COVID-19 pandemic on “Hospital/Medical Institutions,” “Individual(s),” and “Other(s)” channels. Secondary aims are to investigate if different categories of YouTube channels produce more accessible fitness content and highlight any disparities in disability-friendly PA content on YouTube.

**Methods:**

A cross-sectional text analysis of exercise-related YouTube videos was conducted. The authors used Python (version 3.0) to access the YouTube database via its data application programming interface. Terms pertaining to PA that were searched on YouTube were *at-home exercise*, *exercise at home*, *exercise no equipment*, *home exercise*, *home-based exercise*, *no equipment workout*, and *workout no equipment*. Various elements (eg, view count and content generation) of the videos published between January 1 and June 30, 2019 (n=700), were compared to the elements of videos published between January 1 and June 30, 2020 (n=700). To capture a broad idea of disability-friendly videos on YouTube, videos were labeled “accessible” if they were found in the first 100 video results and if their title, description, or tags contained the following terms: *para*, *paralympic*, *adaptive*, *adapted*, *disabled*, *disability*, *differently abled*, *disability-friendly*, *wheelchair accessible,* and *inclusive*. Each video and channel were categorized as “Hospitals/Medical Institutions,” “Individuals,” or “Other(s).”

**Results:**

The analysis revealed a statistically significant increase in viewership of fitness content on YouTube (*P*=.001) and in fitness content generated by Hospitals/Medical Institutions (*P*=.004). Accessible terms applicable to people with disabilities had minimal appearances in 2019 (21 videos) and 2020 (19 videos). None of the top viewed fitness videos that populated on YouTube from 2019 or 2020 were accessible.

**Conclusions:**

The proportion of accessible disability-friendly videos remains diminutive relative to the prevalence of disability in the general population, revealing that disability-friendly videos are seldom findable on YouTube. Thus, the need for disability-friendly fitness content to be easily searched and found remains urgent if access to digital fitness resources is to improve.

## Introduction

### Background

Physical activity (PA) is a critical health strategy for the prevention and maintenance of strong physical and mental health as well as upholding a high quality of life [1-4]. There is strong evidence that people with disabilities report markedly lower rates of PA than their abled-bodied peers [1-4]. For example, according to one study, only 45% of Americans adults with a mobility disability participated in aerobic PA [5]. This lower rate of PA, in part, explains how people with disabilities present with serious illnesses such as obesity, heart disease, stroke, diabetes, and cancer at higher rates than the general population. Therefore, strategies to address these barriers should be developed [1].

People who are disabled have often struggled to access exercise trainers and equipment due to a lack of social support in the fitness and sports sectors, insufficient knowledge of disability among fitness instructors, and a shortage of adaptive fitness resources in gyms [3,4,6]. For example, Richardson et al [4] conducted semistructured interviews with individuals with disabilities about their experiences with the gym. Although several participants indicated that they believed the gym could have the power to improve their physical wellness and social engagement, they also noted that their experiences were often at odds with the gym’s culture [4]. This proves to be an extreme setback, as it has been documented that people with disabilities tend to be more willing to participate in PA if the gym instructor has medical knowledge of their particular diagnosis or disability [3,4,7]. In fact, one study conducted by the Lakeshore Foundation in collaboration with Degree found that 81% of people with disabilities feel uncomfortable using traditional gym and fitness spaces and resources [8]. The reasons for this include, but are not limited to, having greater trust in the source of instruction and greater comfort in the safety of PA if it is being led by someone who would understand the manifestations and possible limitations of a particular diagnosis [7]. These barriers will require systemic change. In the interim, it is possible that people with disabilities might consider alternative modes of PA, such as accessing digital fitness resources.

Understanding the accessibility of fitness resources for people with disabilities on social media platforms such as YouTube could be beneficial for them since some people with disabilities who have discomfort toward in-person fitness settings might be more inclined to use online resources. Thus, considering the need for at-home PA resources due to the social deterrents associated with in-person PA for some people with disabilities, investigations into the accessibility of digital fitness resources as an alternative for people with disabilities are timely and warranted.

Furthermore, the COVID-19 pandemic has increased our dependencies on digital options for activities such as fitness [9-13]. Though increases in PA content on YouTube have not been widely reported, individuals and groups of people creating fitness content on YouTube have seen significant spikes in metrics of engagement with digital resources, such as their number of subscribers and views [14]. For example, more patients have begun relying on hospitals’ or medical institutions’ online fitness sessions to improve their stress and anxiety [15]. Research has found that not only can engaging in PA online be effective in providing the same benefits of more traditional modes of PA [16] but also transitioning to the virtual space has resulted in some benefits, one being the larger audience with whom fitness instructors interact [17,18]. An example of this includes The University of Milan’s *#StayHomeStayFit* movement. This movement reached over 21,000 people, which is a 100-fold increase compared to their prior in-person fitness classes [19]. Given the increasing popularity of digital PA resources, it is important to determine if their accessibility has extended to the disability community as well. Moreover, the unique barriers to in-person PA resources for people with disabilities make investigations into the accessibility of digital fitness resources for people with disabilities, and how these resources could be extended in a postpandemic world, important.

Although there could be a shortage of digital PA resources for people with disabilities, the authors acknowledge that such content may exist on YouTube as well. Our principal concern is how easily this content can be discovered for use when general terms related to fitness are used. If a person with a disability spends a disproportionate amount of time searching for accessible videos or cannot successfully identify it, they are not having an equitable experience to that of their abled-bodied peers. Therefore, the existence of disability-friendly content—content that is created for or adaptable to people with disabilities—is not the focus of this study. Instead, the authors are investigating whether disability-friendly content can be easily found using common search terms.

### Prior Work

Many studies have been conducted on YouTube videos, but few have analyzed the accessibility of YouTube videos for people with disabilities. Most prior work concerns the accessibility of physical fitness centers for people who are disabled, not the accessibility of online fitness content [20-22]. Thus, to the authors’ best knowledge, no studies have analyzed this matter.

### Objectives

This study has two primary objectives. The first is to assess how frequently disability-friendly accessibility terms are used in YouTube fitness videos when users search general PA terms. The second primary objective is to determine if there were changes in the accessibility of disability-friendly PA resources on YouTube between before and during the COVID-19 pandemic. The secondary aims are to ascertain if certain types of channels produce more accessible fitness content and to highlight disparities in accessing fitness opportunities on YouTube for people with disabilities, if any exist.

## Methods

### Video Collection for Study Analysis

A cross-sectional text analysis of exercise- and fitness-related YouTube videos was conducted. Data about videos published from January 1 to June 30, 2019 (“pre-pandemic”) and from January 1 to June 30, 202l (“during COVID-19 pandemic”), were collected using the following search PA terms: *at-home exercise*, *exercise at home*, *exercise no equipment*, *home exercise*, *home-based exercise*, *no equipment workout*, and *workout no equipment*. The authors selected these terms to capture broad exercise content (eg, “exercise” and “workout”) that could be used without the need for equipment most people only can access at a gym. The authors recognize that other fitness terms could be used, such as terms referring to a specific sport (eg, “basketball”), but this investigation aims to capture the experience of using YouTube for PA for the general public rather than for smaller groups of individuals who play specific sports. General terms related to fitness were searched instead of specific terms related to people with disabilities, as the study’s purpose is to determine whether mainstream PA videos include accommodations for people with disabilities. January 2020 was deemed a starting date for COVID-19 videos given that this is when the first lockdown in the world was reported [23].

In lieu of a random sample, we sought to replicate the YouTube video search process to make practical conclusions about the experience of finding accessible videos on YouTube. Prior work suggests that 95% of YouTube traffic is on the first page of search results, which contains 20 to 30 videos [24]. Thus, to establish a rigorous sample size that captures the videos most viewers access, this study collected the first 100 videos that populated on YouTube for each PA search term in both 2019 and 2020. Videos were eligible for inclusion regardless of the country of its creation if the video was created in the English language so that they would be searchable to the study investigators. The authors used Python (version 3.0; Python Software Foundation) to access the YouTube database via its data application programming interface [25]. To deidentify the collected data, YouTube channels and videos were labelled as channel or video as “1, 2, 3...”

### Defining “People With Disabilities” and “Disabled Individuals”

These two terms are used interchangeably to reflect a balanced use of disability-friendly language. For the purpose of this study, people with disabilities refers to any individual who self-identifies with a physical, psychological, or intellectual disability. Since this is a preliminary investigation into disability-friendly content on YouTube, the authors are not framing the definition of disabled individuals around a specific diagnosis or criteria. The authors are more concerned with whether the yielded content includes accessibility terms, and less so with whether the people searching for these terms have a particular disability.

### Measuring “Accessibility”

Although there may be videos on YouTube that contain disability-friendly content, if they cannot be efficiently found, their utility to people with disabilities diminishes. Therefore, for the purposes of this preliminary assessment of accessible PA videos on YouTube, a video was deemed “accessible” if it was found in the first 100 results from the PA search terms and if its title, tags, or description contained one of the following accessibility terms: *para* *paralympic*, *disabled*, *disability*, *differently abled*, *disability-friendly*, *wheelchair-accessible*, *adaptive*, *adapted*, or *inclusive*. These terms were not used in the initial search for PA content since the investigators wanted to ascertain how common accessibility terms are used within commonly searched and viewed PA videos.

The authors acknowledge that videos meeting these criteria still may not be accessible to all users and that additional terms may be appropriate. However, the authors agreed that the aforementioned terms were appropriate and should be analyzed for the following reasons. *Para* or *paralympic* have an implication that combines disability-identifying individuals with sport or fitness [26]. The terms *disabled*, *disability*, *differently abled*, and *disability-friendly* were selected since they are all centered around the word “disability.” It should be noted that although the authors will not use the term *differently abled* to refer to people with disabilities since the term is generally opposed within the disability community [27], it is still frequently used and therefore should be searched to better ascertain what terms content generators might use to describe disability-friendly content [28]. The term *wheelchair-accessible* was included since many individuals who are disabled use wheelchairs, and all these individuals face similar challenges in accessing fitness resources [21]. *Adaptive*, *adapted*, or *inclusive* were analyzed because, although as stand-alone terms they do not necessarily denote disability-friendliness, when combined with words associated with PA, the connotation becomes stronger. The concept of inclusive sport and fitness has shaped an association with disability [29,30].

### Parameters Collected From Videos

Since video titles and YouTube channel names alone often do not provide comprehensive descriptions of video content, video tags (words or phrases creators choose with which to associate their videos) and video descriptions were also gathered and analyzed. Frequencies of the appearance of “accessible” terms in video tags, descriptions, and titles were recorded.

### Data Analysis

Words with the greatest frequencies of appearance in the video titles, tags, and descriptions were collected to assess potential differences in how content generators were describing and tailoring their videos, and if accessibility terms were among the words with greatest frequency. Other collected metrics were compared between 2019 and 2020 content, including the view counts of the generated videos. Frequent consecutive wording pairs (“bigrams”) were compiled within the included video using the tidytext package (version 0.3.1) in R (R Foundation for Statistical Computing) because bigrams can be programmed to remove extraneous words such as “or” and “the” that do not speak to unique context or PA YouTube videos, thereby giving better insight into the video’s specific content. For example, the words “Yoga for neck pain” would generate “yoga” and “neck” as one bigram and “neck” and “pain” as another bigram. If a hyperlink or name was generated in bigrams, it was replaced with the annotation “[hyperlink],” “[omitted first name],” or “[omitted last name].”

### Categorization of Channels

Each video and channel was categorized based on whether the channel is run by “Hospitals/Medical Institutions,” “Individuals,” or “Other(s).” Videos published on a hospital’s or medical institution’s channel were categorized separately from videos created by individuals—a singular person unaffiliated with an established hospital, medical institution, or practice. Remaining videos were placed in the “Other(s)” category. Examples include a certified hospital being placed in the “Hospitals/Medical Institutions” category, a singular person creator being placed in the “Individual” category, and a group creator placed in the “Other” category. This categorization provided greater insight into which content generators are producing the most accessible disability-friendly exercise content on YouTube before and during COVID-19. Attention was given to the "Hospitals/Medical Institutions" channels given that people with disabilities report greater comfort with receiving PA instruction from medical professionals [4]. The creditability of the “Individual(s)” channels, however, cannot be verified with as much ease. Therefore, people with disabilities may be reluctant to use them as frequently for PA. Further, given their expertise with people with disabilities, hospitals and medical institutions might be more inclined to implement accessible terms when posting PA resources during COVID-19 [8,15]. The initial text analysis (eg, search terms such as *exercise no equipment*) and the subsequent text analysis for disability-friendly terms (eg, search terms such as *adaptive*) were compared across the categories including a chi-square test on the change in video generation by "Hospitals/Medical Institutions" channels between 2019 and 2020.

## Results

Video titles, descriptions, tags, and transcriptions were collected for a total of 1400 PA videos between 2019 and 2020. The 1400 videos include the first 100 videos to populate for each of the seven PA search terms created in each year (2019 and 2020). Removing duplicate videos resulted in 1038 unique videos (508 in 2019 and 530 in 2020). Viewership in 2020 of content created in 2020 increased significantly when compared to the viewership in 2019 of content created in 2019 with median video view counts of 52,288 (IQR 2891-401,879) and 122,837 (IQR 7257-728,854) for 2019 and 2020, respectively (*P*=.001).

The analysis revealed that accessible terms applicable to people with disabilities had minimal appearances in 2019 (21 videos) and 2020 (19 videos) among the 1038 unique videos.

**Figure 1:**
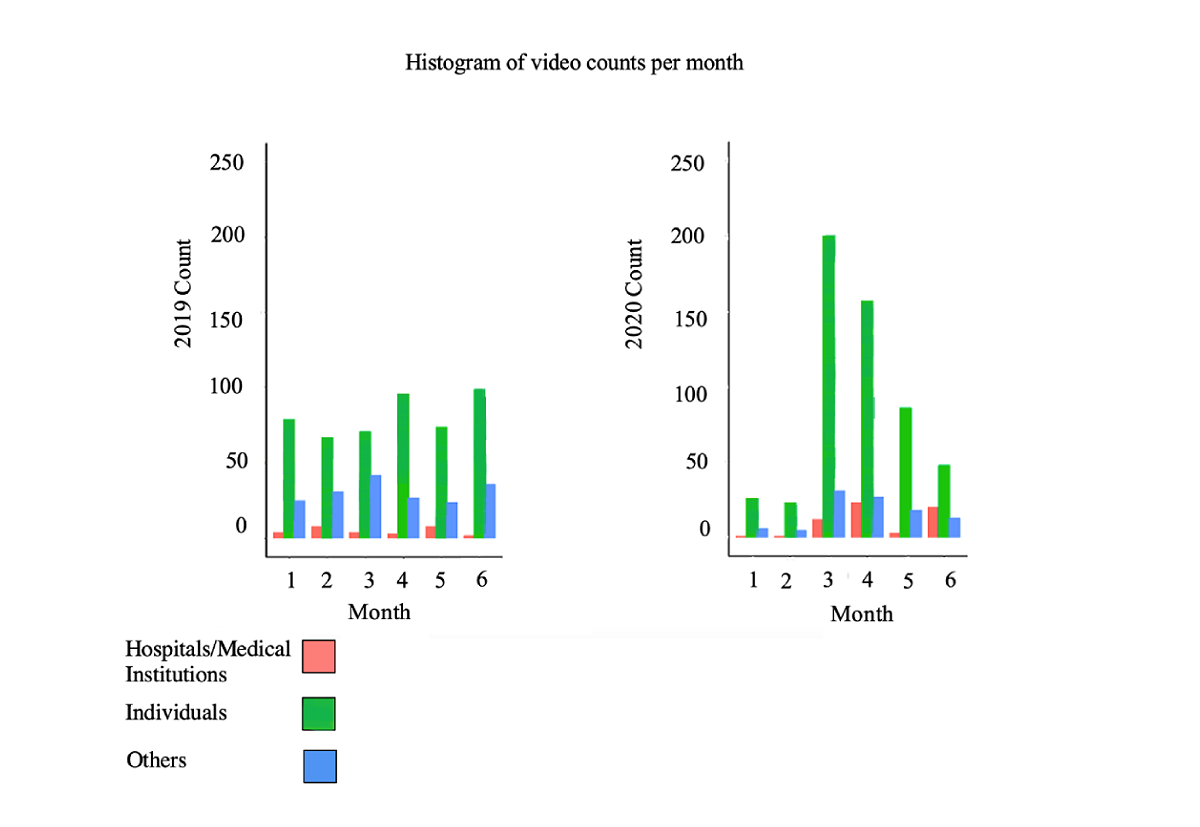
Histogram of total videos published from January to June in 2019 and 2020.

Considering the three domains of interest, “Individuals” channels generated more exercise-related videos between January and June 2020 when compared to 2019. The videos created by each category are summarized in Figure 1. Among the top 10 fitness content generators on YouTube with the most views on their videos, none were "Hospitals/Medical Institutions" channels in 2019, and one was a "Hospitals/Medical Institutions" channel in 2020 (Table 1). After accounting for 29 of the 508 (6%) unique videos from 2019, "Hospitals/Medical Institutions" channels generated 60 of the 530 (11%) videos from 2020 (Figure 1). To investigate whether the proportion of the most viewed videos created by "Hospitals/Medical Institutions" channels had increased significantly in 2020, a chi-square test was conducted that revealed a moderate but statistically significant increase (5%) in PA content generated on YouTube by "Hospitals/Medical Institutions" channels (χ²=8.1476; *P*=.004).

**Table 1:** Top 10 fitness channels on YouTube in 2019 and 2020 (based on video views).

Deidentified channels (2019)	Channel view count as of June 2020 (rounded to nearest million)	Category (2019)	Deidentified channels (2020)	Channel view count as of June 2020 (rounded to nearest million)	Category (2020)
1	29	Individuals	1	34	Individuals
2	28	Others	2	28	Individuals
3	25	Individuals	3	22	Individuals
4	17	Individuals	4	21	Hospitals/Medical Institutions
5	14	Individuals	5	18	Individuals
6	13	Others	6	15	Individuals
7	13	Individuals	7	13	Individuals
8	13	Others	8	12	Others
9	13	Individuals	9	12	Individuals
10	13	Individuals	10	10	Others

When the PA terms were searched, none of the study’s accessible terms populated in the 20 words with the largest aggregate word counts or the top 10 most frequently used word pairs (bigrams) for 2019 and 2020 (Tables 2 and 3). In 2020, two bigrams were tied in 10th place resulting in 11 bigrams being reported.

All the top five viewed videos created by “Hospitals/Medical Institutions,” “Individuals,” and “Other(s)" channels in 2019 and 2020 were inaccessible (Table 4).

**Table 2:** Top 20 words with largest aggregate word count of 1400 video descriptions in 2019 and 2020 (excludes filler words).

Year and word		Word appearances, n
**2019**		
	workout	139
	home	88
	https^a^	46
	body	42
	exercise	42
	video	38
	minute	30
	5	27
	Fat	26
	equipment	25
	exercises	24
	abs	23
	10	20
	free	20
	http	20
	hiit	18
	min	18
	cardio	15
	download	15
	visit	15
**2020**		
	workout	150
	home	109
	https	56
	body	41
	video	35
	minute	34
	5	32
	exercise	32
	equipment	27
	fat	26
	free	26
	abs	25
	ready	23
	hiit	22
	min	22
	exercises	21
	10	20
	burn	19
	visit	19
	join	18

^a^Before tokenization (text parsing), symbols are converted into white space. Accordingly, *http* was kept in the word tally after symbol removal from any embedded link in the video description.

**Table 3:** Top 10 bigram counts of 1400 video descriptions in 2019 and 2020.

Year and bigram			Bigram appearances, n
**2019**			
	home workout		19
	visit https		15
	https [hyperlink]		13
	body workout		12
	home exercise		12
	abs workout		11
	5 minute		9
	10 minute		8
	cardio workout		8
	[omitted first name] [omitted last name]		8
**2020**			
	home workout	21	
	visit https	19	
	https [hyperlink]	18	
	abs workout	17	
	[omitted first name] [omitted last name]	14	
	5 minute	13	
	join [omitted first name]	13	
	body workout	12	
	home exercise	10	
	10 minute	8	
	body home	8	

**Table 4:** Top five fitness viewed videos by Hospitals/Medical Institutions, Individuals, and Others in 2019 and 2020.

Year, category, and deidentified video titles				Accessible or not accessible		View count as of June 2020, n
**2019**						
	**Hospitals/Medical Institutions**					
		1	Not accessible		857,433	
		2	Not accessible		400,604	
		3	Not accessible		325,860	
		4	Not accessible		320,632	
		5	Not accessible		308,392	
	**Individuals**					
		1	Not accessible		31,972,886	
		2	Not accessible		23,421,718	
		3	Not accessible		23,421,342	
		4	Not accessible		23,418,009	
		5	Not accessible		23,292,783	
	**Others**					
		1	Not accessible		29,604,801	
		2	Not accessible		29,600,357	
		3	Not accessible		29,414,241	
		4	Not accessible		12,927,417	
		5	Not accessible		12,924,144	
**2020**						
	**Hospitals/Medical Institutions**					
		1	Not accessible		3,108,295	
		2	Not accessible		1,708,937	
		3	Not accessible		58,240	
		4	Not accessible		44,078	
		5	Not accessible		14,616	
	**Individuals**					
		1	Not accessible		24,521,523	
		2	Not accessible		22,420,881	
		3	Not accessible		19,890,094	
		4	Not accessible		19,484,086	
		5	Not accessible		15,835,194	
	**Others**					
		1	Not accessible		18,331,311	
		2	Not accessible		3,589,580	
		3	Not accessible		2,900,454	
		4	Not accessible		2,251,818	
		5	Not accessible		2,174,462	

## Discussion

### Significance of Findings

Although there was a statistically significant increase in the number of videos created by "Hospitals/Medical Institutions" channels in the top viewed videos over the study period, none of the videos by these creators were accessible by our study’s definition. The authors created their definition of accessible by considering that *disability* encompasses visible impairments such as amputations and invisible disabilities like chronic pain and disease [31-33]. Before the pandemic, many people with disabilities would have benefitted from digital resources since they struggled to find adequate fitness programs due to barriers such as inaccessible buildings, classes, and equipment, or cost and limited social inclusion [6]. During the pandemic, the need for digital resources was heightened due to lockdowns. The statistically significant increase in views on PA videos on YouTube during COVID-19 reflects the increased dependency on digital resources during the pandemic. The absence of a proportional increase in videos using the study’s accessibility terms, however, reinforces the need for YouTube content to be more accessible for people with disabilities and that higher viewership does not necessarily correlate with greater utility.

As full participants in this active social media platform [34], persons with diverse disabilities and ailments could benefit from popular YouTube channels including accommodations for people with disabilities. Although more content curated for or adaptable to people with disabilities may exist, it is unfair that disabled individuals have barriers to accessing these digital resources with comparable ease, especially given that over 1 billion people have a disability globally [35]. YouTube content creators are encouraged to include some accommodations for people with disabilities in their PA videos to make fitness a more inclusive environment.

The analysis of the most frequently used 20 words and word pairs in video descriptions showed no words applicable to people with disabilities, even after reviewing the list for potentially relevant terms not part of the study’s accessible terms. This suggests that most of the content created for at-home exercises were either not inclusive of people who are disabled or would be quite difficult for people with disabilities to find. When terms that could be relevant to disabled users are used by creators less frequently, the question of whether disability-friendly content exists becomes less significant than the question of if such content can be found for use. Not being in the first 100 results shown by YouTube means that the content will rarely be accessed because users rarely look beyond the first page of results.

This analysis shows that the standards for giving everyone equal access opportunities are not being met. In this sense, the COVID-19 crisis has further exposed and exacerbated pre-existing social inequities such as disability stigma and ableist attitudes [36-38]. A particularly damaging form of ableism is the reality that people who are disabled are often invisible to mainstream citizens, programs, and policies. Despite the global burden of disability, for instance, even sweeping international policies have been called out for omitting and failing to consider the experiences of people who are disabled [39].

Beyond YouTube, however, it is encouraging that the COVID-19 pandemic has accelerated action in grassroots and international advocacy groups, as they increasingly recognize the imperative need for digital inclusiveness—including with exercise, health, and fitness content. Mooven, an online resource center, was created in response to the stay-at-home orders. With the help of the International Federation of Adapted Physical Activity, Mooven offers guidance and feedback on exercises [40]. Additionally, the nonprofit Inter Campus uses sports to develop resilience in children and help them cope through the pandemic. On the European front, many programs are taking action to adequately prepare trainers to work with people with disabilities [41]. For digital media access, the Universal Fitness Innovation & Transformation organization created a repository of fitness content specifically for people who are disabled and persons with chronic pain [42]. Finally, in regard to overall connectivity, a United Nations Children’s Fund (UNICEF) program increases internet connection for children in 11 different countries [43]. These programs’ work to increase outreach provides a positive outlook on the increased accessibility of sports.

### Limitations and Next Steps

This preliminary assessment of the availability and searchability of disability-friendly fitness videos on YouTube has several limitations that could be addressed through subsequent studies.

The first limitation is selection bias of search terms. The authors sought to select sensible terms that were conducive to both exercise and disability-friendly content. This approach did populate videos focused on specific disabilities. The investigators are aware, however, there are other terms that could fulfill the same purpose and, therefore, all potentially relevant terms were not included in this study. As acknowledgement of this, the investigators consider this study a preliminary study focusing on accessible fitness content on YouTube for disability in general. Similar studies in the future could search for fitness content targeted for patients with specific conditions such as “stroke,” “cerebral palsy (CP),” “multiple sclerosis (MS),” and “rheumatoid arthritis” to provide the opportunity to search for more targeted results. Additionally, cross-sectional methodology introduces inherent limitations related to generalizability. To manage this bias, the investigators selected a point in time where there was a reasonably suspected shift in video curation on YouTube given the COVID-19 pandemic.

This study solely sought to assess how a generic YouTube user could find disability-friendly content. In other words, the investigators cannot conclude from this investigation if people with disabilities find the videos useful or the proportion of viewers that people who are disabled represent. A future study that would be merited would be a qualitative study into the experiences of people with disabilities using YouTube as a fitness resource. Given the cited preference among some people with disabilities for content created by relevant experts [8] and the fact that the overall increase in fitness content created by "Hospitals/Medical Institutions" channels on YouTube during COVID-19 did not translate to a meaningful increase in accessible content, another future study would include analyzing accessibility of disability-friendly videos only created by Hospitals/Medical Institutions. Members of the disability community have previously identified safety as an important variable in their decision to engage in PA [44,45]. Although it is possible for “Individual” or “Other” channels to provide safe PA options for people with disabilities, not all “Individuals” will have experience working with people with disabilities unlike "Hospitals/Medical Institutions." If safety is a key determinant of engagement, people with disabilities may be dissuaded from participating. Thus, understanding the amount of content that "Hospitals/Medical Institutions" channels create can provide further insight into the accessibility of exercise videos available on YouTube.

Another limitation is that the official beginning of lockdowns varied per country. This may have affected the quantity of content created during each month analyzed in this study. Despite this, there were lockdowns as of January 2020, which justifies this choice for the study. A future study could analyze fitness videos created only in certain countries and limit the search to the dates where lockdowns were in place in that country.

Videos analyzed for this project were limited to being in English so they could be searchable to the study investigators. This may have resulted in videos created in different languages that could be useful for people with disabilities being excluded from this study. To get a more comprehensive understanding of all PA videos, a future study could analyze videos created in multiple languages.

### Conclusions

This study concludes that current YouTube general fitness content is either lacking in disability-friendly content or the content is not easily accessible for YouTube users who are disabled. Despite COVID-19 galvanizing a broader appreciation for seeking PA digitally, this YouTube analysis found there were no increases in accessible disability-friendly exercise content since the pandemic, including Hospitals/Medical Institutions, which have been a source of trust and engagement for the disability community. Increasing disability-friendly fitness content will be important for improving barriers to digital fitness resources within the disability community in a post–COVID-19 era.

## Abbreviations

CP: cerebral palsy
MS: multiple sclerosis
PA: physical activity
UNICEF: United Nations Children’s Fund
